# “I Think I Could Have Used It Better”: Experiences of Youth with High HbA1c Commencing Advanced Hybrid Closed-Loop Therapy in a Clinical Trial Setting—A Qualitative Research

**DOI:** 10.1155/2024/6260002

**Published:** 2024-09-03

**Authors:** Alison Roberts, Julie Dart, Selena Lloyd, Keely Bebbington, Janice M. Fairchild, Geoffrey R. Ambler, Fergus J. Cameron, Elizabeth A. Davis, Timothy W. Jones, Mary B. Abraham

**Affiliations:** ^1^ Children's Diabetes Centre Telethon Kids Institute The University of Western Australia, Perth, Australia; ^2^ Department of Endocrinology and Diabetes Perth Children's Hospital, Perth, Australia; ^3^ Murdoch University of Western Australia, Murdoch, Australia; ^4^ Department of Endocrinology and Diabetes Women's and Children's Hospital, Adelaide, Australia; ^5^ Institute of Endocrinology and Diabetes The Children's Hospital at Westmead The University of Sydney, Sydney, Australia; ^6^ Department of Endocrinology and Diabetes Royal Children's Hospital, Melbourne, Australia; ^7^ Discipline of Paediatrics Medical School The University of Western Australia, Perth, Australia; ^8^ Centre for Child Health Research The University of Western Australia, Perth, Australia

## Abstract

**Background:**

Advanced hybrid closed-loop (AHCL) therapy improves glycemia. However, it is not known if there is an improvement in overall outcomes with AHCL for youth with type 1 diabetes (T1D) at high risk of diabetes-related complications. The study aimed to capture the experiences of youth with suboptimal glycemic control when commencing AHCL therapy in a clinical trial setting.

**Methods:**

This was a singlecenter substudy of a multicenter 6-month randomized clinical trial. Youth between 12 and 25 years of age on insulin pump therapy with HbA1c > 8.5% (> 69 mmol/mol) who commenced AHCL therapy with Medtronic MiniMed™ system were invited to participate in a semistructured interview after 6 months of AHCL. Open-ended questions were used to explore the participants' lived experience of AHCL in improving their glucose levels and its impact on diabetes management and well-being. The interviews were audiorecorded, transcribed, and analyzed using thematic analysis.

**Results:**

Ten youth with T1D with a mean (SD) age of 17.4 (2.9) years, diabetes duration 10.7 (4.8) years, HbA1c 10.2 (0.8)%, or 87 (9.5) mmol/mol at enrollment participated in the interview. Three main themes were identified: (1) improved glycemia despite not using closed loop to its full potential; (2) persistent diabetes burden; and (3) a need for increased psychosocial and clinical support. Although improved glycemia was noted with AHCL therapy, participants reported ongoing motivation issues and used the system suboptimally. They continued to experience distress with overall diabetes management and acknowledged the need for ongoing support from family and health professionals.

**Conclusion:**

All participants reported overall satisfaction with improved glucose levels, however, the persistent diabetes burden impacted their ability to use AHCL optimally. The need for ongoing monitoring with support and interventions to enhance psychological care remains vital for youth with suboptimal diabetes management.

## 1. Introduction

Advanced hybrid closed-loop (AHCL) therapy is the recommended management for youth with type 1 diabetes (T1D)[1]. The algorithm-driven insulin delivery has unequivocally established superior clinical outcomes to previous therapies, both in clinical trials[2, 3] and real world studies[4, 5]. There is also an increasing interest in addressing the impact of AHCL on psychological outcomes in youth with T1D [6]. Qualitative studies suggest that AHCL may improve psychosocial well-being and reduce the burden associated with diabetes management[7, 8]. However, this data have typically been collected as part of clinical trials involving participants who tend to be proactive in their diabetes management and have generally acceptable glycemia. These studies have reported increased confidence, flexibility, and independence in diabetes management in youth with long duration of diabetes [9] and in youth and their parents who were commenced on AHCL after diagnosis [7, 10]. However, less is currently known about the experiences of youth with very high HbA1c using AHCL.

Adolescence is a period of significant physical and psychosocial development, and the additional need of diabetes management can lead to youth often not meeting the recommended targets of glycemia. Less than a quarter of youth achieve the recommended HbA1c of <7% (53 mmol/mol) with more than a third with high HbA1c > 9.0% (75 mmol/mol)[11]. The impact of AHCL therapy in this vulnerable group with high HbA1c has been explored in a few studies wherein participants transitioned from MDI to the Medtronic 780G AHCL. These studies showed an improvement in glycemia [2, 12, 13] along with an improvement in quality of life and diabetes treatment satisfaction [12]. We had the opportunity to explore these outcomes in a 6-month randomized clinical trial that investigated the impact of AHCL on glycemic and psychosocial outcomes in 42 youth who continued to have suboptimal glycemia on insulin pump therapy. The cohort had a mean HbA1c of 10.3% (89 mmol/mol), and 71% of this group also exhibited significant diabetes distress (Problem Areas in Diabetes (PAID) score > 40). There was an improvement in HbA1c with AHCL (mean adjusted difference between two groups of −8.4 mmol/l). However, there was no improvement in participant-reported outcomes (PROs; diabetes distress, quality of life, anxiety, depression, and fear of hypoglycemia) between the two groups at the end of 6 months of study [14]. To further explore and gather in-depth information regarding the experiences of participants using AHCL for 6 months, the lead site undertook a qualitative substudy.

## 2. Methods

This qualitative substudy used a phenomenological approach to explore participants' individual experiences[15]. Participants were recruited from the 6-month multicenter randomized clinical trial in Australia which evaluated the glycemic and psychosocial outcomes of AHCL compared to standard continuous subcutaneous insulin infusion (CSII) with or without continuous glucose monitoring (CGM). The trial was prospectively registered with the Australian New Zealand Clinical Trials Registry (ACTRN 12619001452189), and ethics approval was received at each site. Participants in this study were aged between 12 and 25 years, with T1D for more than a year, with mean and most recent HbA1c > 8.5% (> 69 mmol/mol) on CSII ± CGM. Participants in the control arm continued their usual treatment, while participants in the intervention arm used the AHCL algorithm in the Medtronic MiniMed™ insulin pump for 6 months. After 6 months, all participants in both groups had the opportunity to use AHCL for another 6 months. All 11 participants who used AHCL for 6 months at the lead site were invited to participate in a semistructured interview. Consent for participation in the study was taken by the research nurse at the start of the study.

### 2.1. Device

The study used the investigational MiniMed™ 670G Version 4.0 AHCL pump (Medtronic, Northridge, CA, USA) equivalent to the MiniMed™ 780G system (without Bluetooth connectivity) and switched to the MiniMed™ 780G insulin pump in October 2021. Participants used the Guardian™ Sensor 3 and transmitter during the study.

### 2.2. Data Collection

Interviews were completed at the final study visit which occurred between February 2021 and September 2022. An interview guide (Table 1) designed by the clinical and research team which remained constant during the interview was used to explore participants' experiences managing their diabetes and how they used AHCL. Parents could sit in the interviews as non-participants. Interviews were completed by the same research nurse experienced in conducting qualitative interviews (AR), who was neither part of the larger RCT nor involved in their clinical care.

All participants who used AHCL at the lead site were invited to complete an interview, to capture a wide range of experiences; however, one participant declined due to time constraints. The intention of this substudy was only to interview participants at the lead site, so saturation was not a consideration. The sample size of ten, however, is thought to have been adequate to provide specific data from a range of personal experiences. The specific aim of the research question was narrow, and the quality of interview dialogue was satisfactory [16].

### 2.3. Data Analysis

All interviews were transcribed by SL, deidentified, and checked by two researchers (AR and SL) for accuracy. One experienced researcher and a student researcher read and reread all transcripts and independently hand-coded all transcripts. Using a framework analysis approach, codes were transferred to an Excel sheet, and data was added from the transcripts. This process was completed independently by both researchers. This is a systematic and flexible approach used to manage and analyze large amounts of qualitative data and is appropriate when working with novice researchers as part of a team [17]. The framework analysis uses seven stages to summarize data through transcription, familiarization, coding, developing an analytical framework, applying and charting data into the framework, and interpreting the data. During the analysis phase, AR and SL met regularly to consider and consolidate codes and develop themes. To ensure credibility, this data were presented to the whole research team for discussion, and final themes were determined which represented the overall data. A detailed journal was kept providing dependability and confirmability of the process.

## 3. Results

Fifteen youth participated in the main trial at the lead site with four withdrawals (two each in the control and AHCL groups). Withdrawal reasons cited in the AHCL group were mental health issues and study burden. Hence, in this substudy, there were a total of 11 youth who were eligible and had used AHCL for 6 months. One participant was not able to participate in the interview due to time commitments, while the remaining 10 youth (six from the AHCL group in the primary phase and four from the control group who used AHCL in the extension phase) participated in this substudy.  Table 2 provides the characteristics of the cohort. The mean (SD) age of the cohort was 17.4 (2.9) years (range 13.3–22.5 years), diabetes duration was 10.7 (4.8) years with baseline HbA1c of 10.2 (0.8)% (range 9.1–11.3%) at randomization. Time in range (70 −180 mg/dl) was 39 (16)% during the run-in period with a diabetes distress score of 35.3 (10) evaluated using the PAID questionnaire at enrollment. The interviews lasted on average 28 min (range 16–40). The mean (SD) HbA1c after 6 months of AHCL was 9.1 (1.4)%, and the diabetes distress score was 41.5 (22.2). All participants acknowledged that their diabetes management was inadequate before entering the study. This was attributed to forgetting to bolus, not wearing and/or calibrating their sensor, not performing finger-prick blood glucose readings, behaviors commonly associated with diabetes burnout. Some participants identified this as their primary motivation for enrolling in the study. Others reported that they were motivated by the opportunity to try the newest technology.

*“Up for anything that will make life easier*, *and I was at rock bottom*, *so I needed something to lift me up”*. (Participant 4)

**Figure 1:**
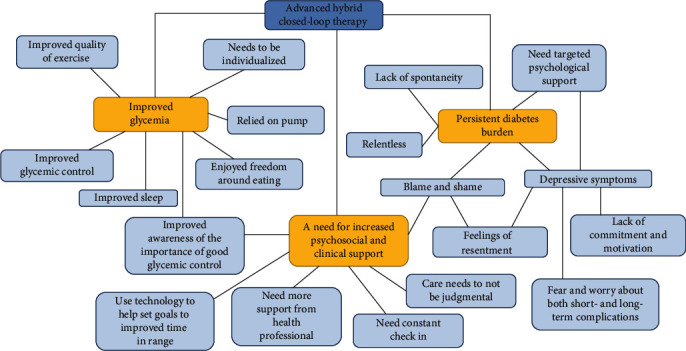
Thematic map.

Three main themes were identified with an overview of these themes provided in Figure 1.

### 3.1. Improved Glycemia—Despite Not Using Closed Loop to Its Full Potential

#### 3.1.1. Improved Glycemia

All participants recognized the benefit they received from AHCL and reported improved glycemia, which included more time in range and reduced hyperglycemia. Participants reported overall improvement in glucose levels, resulting in better energy levels throughout the day, and improved quality of sleep and exercise.

*“Before the study I was probably sleeping more just because my blood sugar levels were not as good and I was a lot more fatigued*, *but I found that while I was on the pump*, *I was able to have less sleep but it was better quality”*. (Participant 5)

*“I had no energy to do anything*, *like at school I would just sit there and like daydream 'cause I just couldn't learn*, *couldn't concentrate”*. (Participant 4)

*“So before I got into the pump*, *I think was around 11*, *but it came down to around 8.6 on my previous HBA1C. Today it's 9*, *but also that's from a couple of other factors like not bolusing and it can definitely be improved*, *but overall*, *it has brought it down well”*. (Participant 7)

Participants reported that AHCL made living with diabetes feel more manageable and noted that this positively impacted their mental health and well-being.

“[*AHCL*] *definitely changed my perspective on it*, *yeah*, *'cause I can see I actually can be…in good range or I can be in good health*, *whereas before I was a bit like*, *ugh*, *I'm always going to feel like this. I think it also impacts my mental health hugely*, *just constantly feeling down and unwell”*. (Participant 3)

*“I absolutely love the auto correction or the auto bolus feature. Like that was just the world saviour for me and I really liked as well with the you know the fact that I could flick across straight away and it would tell me the time that I'd been in range like that night and that day*, *and I found that was a really good positive indicator when it was going well…*” (Participant 5)

Many participants commented that they felt they had more freedom around eating. They were less worried about the kinds of food they were eating, not having to consider every ingredient, and were less likely to bolus for snacks.

*“… there's a bit more freedom involved*, *… you can think about it less and you don't need to consider a lot of factors like fat*, *all of that stuff just slowly seeping in 'cause you know that the automation will eventually get it down”*. (Participant 7)

“*I definitely only just bolus for the big meals”*. (Participant 9)

Participants reported easier management with AHCL with improved quality of exercise due to better glucose levels. Some participants reported the use of a temporary target of 8.1 mmol/l helped with postexercise hypoglycemia. However, other participants reported not using this feature.

*“Used temp target to prevent hypo's post-exercise was very helpful..”* (Participant 5)

“[*AHCL*] *helped improve the quality of exercise that I've had*, *so instead of being high and feeling sluggish while doing it I could be more in range and feel more energetic while doing it”*. (Participant 1)

All participants found AHCL to be valuable and developed trust in the system within weeks. However, some participants did not appreciate the benefits of the system initially and took a few months to make changes to behaviour and management or to wear and calibrate sensors to remain in closed loop.

*“At first it is a bit weird to hand over fully to the pump*, *you're constantly checking on it and seeing what it's doing*, *but after a while you just get to trust it*, *and I don't think there's any way that you could improve that without just*, *time”*. (Participant 7).

*“I was completely fine with it*, *I was happy to just put all my trust in it*, *‘cause yeah*, *it's a pump*, *I've had one for ages so I trust it”*. (Participant 9)

#### 3.1.2. Suboptimal Use of AHCL

Although participants found the system improved overall glucose levels, they acknowledged that they did not use AHCL to its full potential.

*“Like I probably failed the pump a little by not wearing the sensor as much as I should*, *but in that time where I was consistently wearing it was just fantastic”*. (Participant 5)

*“I think that I could use it better*, *but it's just so good…I'm pretty much doing the same thing what I was doing with the other pump and it's so much better*, *like I could go without testing my blood sugar all day and I only need to test when I wake up and when I go to bed and all day*, *I have a blood sugar reading on my pump”*. (Participant 4)

Realizing less effort was needed while in AHCL to maintain glycemia, some participants relied on closed loop to maintain glucose levels without intervention, such as administering additional insulin boluses for meals.

*“I just relied on it* [*closed loop*] *to fix my highs a bit more*, *didn't really work but still relied on it”*. (Participant 1)

*“… I was bolusing when I like first got the pump but then I kinda just got like sick of it and started forgetting when I was eating to like bolus and stuff”*. (Participant 6)

Despite the positive impact on glucose levels, alarm fatigue and interrupted sleep were reported as barriers to technology use, contributing to reduced sensor wear.

*“Alarm fatigue—a massive thing that”s not talked about much—sometimes you just need a break”*. (Participant 5)

### 3.2. Persistent Diabetes Burden

Despite being aware of the potential for long-term complications associated with suboptimal glycemia, participants described struggling with the motivation to engage in diabetes self-management behaviors, particularly in the context of competing priorities in their day-to-day life.

*“I think I could have used it better—why—I just can't be bothered”*. (Participant 4)

*“It* [*diabetes*] *was just less of a priority. I just kind of wanted to shove it to the side for a bit”*. (Participant 7)

*“I struggle with the mental side of it*, *so being motivated to do it every day or continue doing it”*. (Participant 1)

All participants spoke about experiencing diabetes-related distress, attributed to the relentlessness of managing diabetes. Participants spoke about having feelings of resentment, wanting to be like their friends, and anger at the lack of spontaneity.

*“It's really hard to…live life…I don't know*, *everyone else can live life and don't have to worry about their body*, *but…I have to live life…and I have to control my body”*. (Participant 4)

Additionally, some participants mentioned that they were embarrassed to do finger pricks, boluses, or injections in public. They felt people blamed them for their condition and felt judged and continually monitored.

*“It just feels to me that people think that I brought the diabetes on or like something that I did wrong”*. (Participant 4)

### 3.3. The Need for Ongoing Psychosocial and Clinical Support

#### 3.3.1. Support from Family/Friends

All individuals in the study felt well-supported by their family and friends but preferred diabetes to not always be the focus, as they were more than their condition.

*“I didn't like talking about it because it was the only thing*, *we sort of talked about…cause it kind of like goes well*, *you've got diabetes*, *so the only thing about you is diabetes”*. (Participant 1)

Several participants spoke about the worry they cause their parents when their diabetes management was not ideal, which increased the burden of living with diabetes.

#### 3.3.2. Support from Healthcare Professionals

All participants valued the individualized clinical attention and support provided by the research nurse during the study and felt this helped them use the technology well.

*“I don't think I would have used the pump as much as I have throughout the study*, *definitely having the support of them improved it a lot”*. (Participant 7)

Participants acknowledged that they received support from their individual clinics, a few participants; however, commented on the fear of being judged due to the constantly high HbA1c.

*“I don't enjoy clinic appointments just cause sometimes it feels like there is a little bit of judgement from the doctors”*. (Participant 10)

Concern was also expressed regarding the vast differences between pediatric and adult healthcare services mainly due to the lack of support services in the adult healthcare system.

*“Since I aged out of the kids system*, *I went private just because the adult public system is just not very supportive and not very easy to get into*, *or even easy to speak to anyone”*. (Participant 3)

## 4. Discussion

This qualitative study captured the lived experiences of a vulnerable cohort of youth with suboptimal glycemia when commenced on AHCL therapy in a clinical trial setting. The results from the semistructured interviews provide a more detailed and nuanced perspective on the experience of using AHCL, relative to the results of the larger clinical trial, which showed modest improvement in glycemic outcomes with no change in participant-reported outcomes in quality of life, diabetes distress, fear of hypoglycemia and, anxiety, assessed using validated questionnaires. Specifically, it addresses the finding that although AHCL improves glycemia, which was appreciated both objectively and subjectively by the youth, the technology was not used optimally by study participants. Participants continued to experience high levels of diabetes-related distress when using AHCL, which negatively impacted their capacity to engage in self-management tasks necessary to optimally use the system. Participants identified diabetes-related stigma, competing priorities, and reduced motivation as barriers to engaging in diabetes self-management behaviors. Participants voiced the need for ongoing support for their diabetes management from family and/or HCPs. The lived experience of young people with T1D who have suboptimal glycemic levels demonstrates that while AHCL is beneficial in improving glycemia, there remains a significant unmet need for many patients who experience high levels of diabetes-related distress.

Improvement in insulin delivery and glucose monitoring systems has provided the opportunity to improve glucose levels which is observed both in clinical trials and real-life studies with an increasing proportion of users meeting the recommended glycemic targets. Favorable glycemic outcomes have been reported in youth with high HbA1c across various studies[12, 13, 18] which was also acknowledged by the participants during the interview. However, the impact of psychosocial outcomes in this cohort is not consistent. Although there was increased diabetes satisfaction and improvement in quality of life in youth after 12-month AHCL use in those who transitioned from MDI to AHCL[12], there was no improvement in psychosocial outcomes in our RCT which compared AHCL to standard insulin pump therapy. The mean PAID score in youth is 29.3 [14, 19] with a cutoff of 40 [20] used to describe high levels of distress. In our study cohort, the mean score was 35.3 and 41.5 at baseline and 6 months post AHCL highlighting the high levels of distress. A systematic review reported that almost a third of youth experienced substantial distress which is frequently associated with suboptimal glucose levels, low self-efficacy, and reduced self-care[21]. Even with more advanced systems like AHCL, participants struggled to sustain the self-management behavior necessary to use the system optimally. For instance, participants reported improved premeal insulin administration at the start of the study but were not able to sustain this over the study period. Wearing a sensor continuously was an observed barrier in the cohort, especially with alarm fatigue influencing sensor wear. Although international recommendations state that psychological assessments and care should be part of routine diabetes care [22], the availability of psychological care in diabetes centers remains inadequate across centers worldwide [23]. In our study, participants also acknowledged the need for ongoing support and the difficulty in accessing services in the healthcare system.

Our study used the Guardian™ Sensor 3 which required finger-prick glucose testing for calibrations, but this has now been superseded by the Guardian™ Sensor 4 which does not need calibrations. Improved user experiences with reduced burden have been reported from real-world evaluations with transition from G3 to G4 [24]. Improved glucose monitoring devices designed to last longer and have no minimal need for finger-prick blood glucose testing and early introduction of CGM as part of routine care could address some of the barriers and improve sensor wear. Likewise, a fully automated insulin delivery system, capable of dealing with unannounced meals, will reduce the need for user interaction and potentially improve long-term outcomes.

Glycemic outcomes have historically been utilized to characterize the goals of diabetes treatment and have been used as the primary outcome in clinical trials. HbA1c measures were used in the past with our current interest focussed on CGM metrics with international recommendations on reporting established in clinical trials[25]. This focus on glucose levels comes from strong evidence that high glucose levels are a harbinger for future glycation-related long-term end-organ disease[26, 27] and hence the need to improve and optimize glucose levels. Generic and diabetes-specific PRO measures are gathered using standardized questionnaires to evaluate the impact of therapy on their overall health and to gain perception of their overall well-being[28] and reported as secondary outcomes in a few clinical trials. However, the lack of consistency in the PROs used in relation to the questionnaires used and the population to which they are administered, makes interpretation of the findings less generalizable, highlighting the need for unifying international recommendations to guide and inform the design of future studies. Lived experiences gathered using semistructured interviews provide a powerful tool to enrich this information and inform our understanding of the impact of diabetes technologies.

The strength of this study is the methodology with semistructured interviews providing a robust platform to explore the lived experiences of youth. Likewise, this is the first qualitative study addressing the perceptions of a high-risk cohort of youth with high HbA1c and diabetes distress. The interviews were conducted by staff experienced in qualitative research and not related to the clinical trial. Although the interviews lasted for less than 30 min, this was in keeping with our previous work in this age group [9]. The limitations are the small sample size restricted by the number of participants recruited into the clinical trial at a single center. Although the study was able to recruit almost all eligible participants (10/11) who used AHCL for 6 months, data saturation may not have been achieved. The two youth who were on AHCL and withdrew from the study could potentially have added further insights into the study. The interview questions were not designed to detect a change in diabetes burden but to explore participant experiences after 6 months of AHCL use. The study used the Guardian™ 3 sensor which required finger-pricks for calibration and not the Guardian™ 4 sensor, and this could potentially impact the usability of the device. The generalizability of these findings is restricted to a cohort of youth using technology (pump and CGM) with high HbA1c and diabetes distress.

## 5. Conclusion

In conclusion, all participants reported overall satisfaction with improved glucose levels during a 6-month trial of AHCL. However, participants described persistent diabetes-related distress, which continued to complicate diabetes management, highlighting the importance of assessing psychosocial well-being as part of routine care.

## Figures and Tables

**Table 1:** Interview guide.

Tell me about your diabetes-what is diabetes like for you?
May I ask about your glucose control?
Why did you join the study?
Tell me about your sleep/eating/exercise with closed loop?
Does diabetes ever get you down?

**Table 2:** Participant characteristics.

Characteristic		*N* = 10
Age (years) ^*∗*^		17.4 (2.9)

Females, *n*		5

Duration of diabetes (years) ^*∗*^		10.7 (4.8)

Duration of insulin pump therapy (years) ^*∗*^		6.0 (4.7)

Pump, *n*	Medtronic 640 G	5
	Medtronic 670 G	3
	Tandem T slim	2

CGM, *n*	Dexcom G6	8
	Libre	2

HbA1c (%) ^*∗*^	Baseline	10.2 (0.8)
	post-6-months AHCL	9.1 (1.4)

HbA1c (mmol/mol) ^*∗*^	Baseline	88 (8.7)
	post-6-months AHCL	76 (15.3)

Sensor wear time (%)		61.6 (15.9)

Time in closed loop (%)		68.8 (19.3)

PAID ^*∗*^ score	Baseline	35.3 (10)
	post-6-months AHCL	41.5 (22.2)

*Abbreviations*. CGM, continuous glucose monitoring; AHCL, advanced hybrid closed-loop; PAID, Problem Areas in Diabetes;  ^*∗*^mean (SD).

## Data Availability

The interview data used to support the findings of this study are restricted by the Child and Adolescent Health Service Human Research Ethics Committee to protect participant privacy. Data may be available from the corresponding author for researchers who meet the criteria for access to confidential data.
